# On the Adaptive Value of Paranormal Beliefs - a Qualitative Study

**DOI:** 10.1007/s12124-020-09594-5

**Published:** 2021-01-19

**Authors:** Tilmann Betsch, Paul Jäckel, Mareike Hammes, Babette Julia Brinkmann

**Affiliations:** 1 University of Erfurt, Erfurt, Germany; 2 Department of Psychology, University of Erfurt, Nordhäuser Strasse 63, D-99089 Erfurt, Germany; 3 TH Köln (University of Applied Sciences), Cologne, Germany

**Keywords:** Paranormality, Adaptation, Mastery, Self, Meaning

## Abstract

Ten female and five male participants (age range 28–50 years) were recruited at esoteric fairs or via esoteric chatrooms. In a guided face-to-face interview, they reported origins and contents of their beliefs in e.g. esoteric practices, supernatural beings, rebirthing, channeling. Transcripts of the tape-recorded reports were subjected to a qualitative analysis. Exhaustive categorization of the narratives’ content revealed that paranormal beliefs were functional with regard to two fundamental motives – striving for mastery and valuing me and mine (striving for a positive evaluation of the self). Moreover, paranormal beliefs paved the way for goal-setting and leading a meaningful life but, on the negative side, could also result in social exclusion. Results are discussed with reference to the adaptive value of paranormal beliefs.

## Introduction

Paranormal beliefs have survived enlightenment. Despite tremendous advances in science and technology during the past century, many people still believe in magic, astrology, esoterism (e.g., reiki, chakra, aura), supernatural beings (e.g., ghosts, demons), and spirituality (e.g., rebirthing, channeling). In a large, representative German survey (GESIS - Leibniz Institute for the Social Sciences 2013), up to 50% of participants reported believing in the validity of paranormal phenomena and pseudo-scientific healing methods (e.g., anthroposophical healing methods, homeopathy, Bach flowers therapy) to at least some extent. The services and products in the paranormal domain have an estimated annual turnover of more than 15 billion Euros in Germany alone (Klaus 2017; see also Potten and Memminger 2017).

What is the meaning of the term paranormal beliefs? Actually, definitions are diverse (Lindeman and Svedholm 2012). Yet most share a common denominator – the conceptualization of paranormal beliefs as non-religious beliefs about entities, mechanisms, and practices that contradict established scientific knowledge (cf. Betsch et al. 2020; van Prooijen et al. 2017). On the measurement level, however, seemingly diverse beliefs in, for instance, spirituality and magic/witchcraft are highly correlated (e.g., Drinkwater et al. 2017). As such, belief in the paranormal is somewhat like a syndrome. Those people inclined to believe in, say, astrology or spiritual practices are also “open” to many other things that are in opposition to empirical facts (see Betsch et al. 2020, for a discussion). A huge body of psychological research deals with *individual difference*s as potential predictors for paranormal beliefs*.* A plethora of such predictors are documented in the literature, for instance, education and gender (e.g., Aarnio and Lindeman 2005), death anxiety (e.g., Henrie and Patrick 2014; Rasmussen and Johnson 1994), life satisfaction (e.g., Gray and Gallo 2016), thinking style (e.g., Aarnio and Lindeman 2005; Gray and Gallo 2016), numeracy (e.g., Dagnall et al. 2007; Hergovich and Arendasy 2005), probability understanding (e.g., Musch and Ehrenberg 2002; Rogers et al. 2009), ontological confusion (Lindeman et al. 2015), cognitive ability (e.g., Musch and Ehrenberg 2002), and personality (e.g., Henningsgaard and Arnau 2008; Schnell 2012; Williams and Roberts 2016).

In a recent study, Betsch et al. (2020) compared the predictive power of 21 individual-difference variables, which were selected during extensive pretesting. Six factors survived in regression analyses, yielding a model explaining 19% of variance in paranormal beliefs. It consisted of ontological confusion, cognitive ability (negative correlation), openness to new experiences, emotionality, conscientiousness (neg. Corr.) and causality understanding (neg. Corr.). Converging with prior studies, these results show that individual differences are indeed important in accounting for belief variations in paranormality. Notably, however, the six-factor model accounted for only one fifth of the total variance. What is missing? What additional factors account for the remaining 80% of variance?

In this paper, we explore the *functional side* of paranormal beliefs. This may appear odd at first glance. Paranormal beliefs are, by definition, in opposition to established scientific knowledge. However, people presumably seek *accurate* conceptions of the world (Festinger 1954). From an evolutionary perspective, a default presumption is that accurate beliefs are adaptive, whereas misbeliefs are maladaptive (McKay and Dennett 2009). Accordingly, paranormal beliefs, superstition, and magical thinking have regularly been treated as a form of psychopathology that corresponds to deficits in thinking (see Risen 2016, for an overview). The dysfunctional side of belief in the paranormal also plays an important role in clinical psychology through its association with the Diagnostic and Statistical Manual of Mental Disorders (DSM-4®, 1994, category “religious or spiritual problem”, cf. Lange et al. 2000, p. 133). Irwin (1992, 1994) and Wolfradt (1997) discussed the partly dissociative nature of paranormal experiences and their potential linkage to childhood trauma.

In a similar vein, religious beliefs are explained in terms of erroneous reasoning. The standard theory for religiosity assumes that the ubiquity of belief in supernatural beings evolved as an attribution bias, i.e. “to overinfer agency as a means of error management” (cf. Kanazawa 2015, p. 306, for a discussion). In other words, religion is viewed as a by-product of biased thinking. As an alternative approach, Kanazawa (2015) assumes that religiosity reflects an indirect form of adaptation by facilitating positive affect, well-being, and the pursuit of a *meaningful* life. Accordingly, even erroneous or faulty beliefs are assumed to have empowering effects on the individual. The same may be true for paranormal beliefs, as well. Whereas psychologists have been continuously discussing and studying the functional side of religious beliefs for more than 100 years (e.g., James 1902; Gebauer et al. 2012), the adaptive value of paranormal beliefs has received only marginal consideration (but see Houran and Lange 2004; Lange and Houran 1999, who show that paranormal beliefs can reduce fear).

In social psychology, three general motivational principles are considered ubiquitous and of fundamental importance in driving human behavior: (1) striving for mastery, (2) seeking connectedness, and (3) valuing me and mine (Smith and Mackie 2000). The first motivational principle is instrumental with regard to concrete action goals. Utility maximization (Von Neumann and Morgenstern 1947), for example, requires tuning decisions on contingencies and causal relations in the environment. As a second principle, people are assumed to “seek support, liking, and acceptance from the people and groups they care about and value” (Smith and Mackie 2000, p.17). Seeking connectedness is mirrored by a plethora of models such as affiliation theory (McClelland 1987) and the norm-component in attitude-behavior models (Ajzen and Fishbein 1980). The third motive refers to the tendency to valuing me and mine, i.e. to achieve and maintain a positive evaluation of the self. Thus, individuals’ knowledge about their own roles, individual characteristics, and behaviors have a positively biased attitudinal component. In turn, threats to these attitudes will activate defensive goals. Conceptions and strategies are functional to the degree that they eliminate these threats (Kunda 1990).

In this exploratory research, we aim at going deeper into the content of individual stories about paranormality. Our first goal is to examine whether and to what extent spontaneous self-descriptions reveal *functional aspects* of paranormal beliefs. Our second interest relates to whether and to what extent the three motives described above are sufficient to capture the core aspects of these stories.

## Method

### Participants

We recruited ten female and five male participants (age range 28–50) at esoteric fairs and via the internet (e.g. esoteric-related platforms). Participation was voluntary without monetary incentivization. All participants were first informed of the real aim of the study – i.e. understanding the formation of esoteric beliefs by talking with individuals about their personal experiences and views. All participants agreed to their interview being recorded. Information revealing the identity of the person were not recorded or stored along with the coded data.

### Procedure

All participants were interviewed by the same person in a face-to-face situation. In contrast to highly structured approaches, we avoided setting a topical focus (Loosen 2014). Due to the sheer number of different areas of paranormal beliefs, the interviewed persons were free to focus on topics of their own choice. The guide comprised five questions created to elicit anecdotal experiences from participants. These prompts addressed areas of special interests in the field of esoterism, crucial experiences, special biographical aspects in belief formation, role of others, and doubts and criticism. Additionally, the question order was not fixed, which allowed the interviewer to interview the participants as freely as possible. (cf. Kruse 2014, p. 213). Most importantly, we ensured that the interviewer avoided any questions that explicitly addressed potentially functional aspects of paranormal beliefs. Participants were free to terminate the interview at any time. All participants addressed the entire set of questions. The interviews lasted between 5 and 30 min. They were held at either the location of the esoteric fairs, nearby coffee shops, the interviewee’s home or place of business depending on the preference of the interviewed person. Regardless of the circumstances, each interview began with a briefing regarding the study objective, a privacy statement, and the provision of informed consent. The aim of the interview was to access the relevant core aspects of the respective person’s beliefs rather than eliciting elaborate full-life stories. This approach has been shown to be successful even in short interviews. Participants willingly opened themselves to the interviewer, sharing their beliefs, perspectives, and experiences.

## Results

### Coding

Records were transcribed in accordance with standard techniques in qualitative research (Kuckartz et al. 2008, pp. 27 ff.). Goal of the transcription was to make the utterances and statements more readable by focusing on their semantic content. Gross grammatic errors were corrected. Filler words were not adopted. All information that potentially reveals participant identity was eliminated.

### Analyses

We employed qualitative content analysis following Mayring (2002), Schreier (2014), and Stamann et al. (2016). The transcripts were analyzed by three coders. The coders were students of Social Work at the Technical University of Cologne. They were trained in qualitative content analysis and successfully mastered pertinent seminars on qualitative research.

As a first step, all coders together derived super categories from the five prompting questions. As a second step, a sample of five interviews was randomly drawn. Raters assigned content of the transcripts to the super categories and developed refinements of more specific sub-categories in an inductive fashion (cf. Schreier 2014). Analyses on this level aimed at developing a comprehensive category system that covers all of the derived and inductively found contents of the interviews. In a third step, coders separately applied the category system to all interviews. The coding results were re-analyzed by an independent rater (one of the authors) who was so far not involved in the coding process (cf., Mayring and Fenzl 2014, p. 546ff). The evaluation revealed an almost perfect coincidence of coding results across coders. Coders than discussed the few critical cases and decided together about final assignment to categories.

### Category Space

The systematic text analysis yielded five main categories: (1) content, (2) sources, (3) utility, (4) justification, and (5) challenges of the belief. Each category was divided into a number of subcategories that can be accessed in the repository. With the exception of the fifth category, none of these categories directly corresponds to our prompting questions. This shows that the guided interview was effective in eliciting free narratives that go beyond the semantics of the questions. Specifically, participants openly described the *content* of their beliefs in much detail with references to, for example, immaterial entities (e.g. ghosts, angels), spiritual energies, parapsychological/ transcendent practices, and several aspects of religion (e.g., Christianity, Buddhism, Hinduism). The converging observation was that each of the participant’s beliefs could be characterized as a rich schema reflecting an idiosyncratic melting of esoteric, spiritual, and religious motives and themes. In accordance, the second category reflects a rich compilation of *sources*. Mass media, the internet, informal social exchange (e.g., friends), coordinated social exchange (e.g., seminar, quasi-educational means), and one’s own spiritual and paranormal experiences jointly contributed to belief formation. The fourth and the fifth category (*justification, challenges*) could be summarized in terms of dissonance reduction. Participants were aware that some aspects of their beliefs violate common sense and rational thinking. Nevertheless, they sometimes evoked quasi-scientific explanations for justification. Immunization (a belief that is based on one’s own experience does not require further proof) and references to distinguished sources (e.g. “experts”) were common strategies to bolster the belief against multiple challenges. The latter included social influence by a presumed dominance of rationality in society, social isolation, and exclusion in their ingroups as well as a subjectively experienced coercion to hold more socially accepted beliefs (e.g., in terms of Christian dogma). However, participants also mentioned obstacles to their beliefs that originated within themselves such as doubts and the effort to lead a life in accordance with the demands resulting from their beliefs.

### Functional Aspects (Utility)

The third category spontaneously addressed by the participants relates to the utility of their beliefs. Recall the three motivational principles from the introduction: Striving for mastery, seeking connectedness, and valuing me and mine. The first inductively derived subcategory to utility was entitled “Getting along with the world” by the interview coders, who were blind to the research questions (see Table 1). Participants reported several specific functions of their belief such as orientation (goal setting, option identification, and decision making), coping with fear, overcoming critical life events, and explanation (explaining the unexplainable, understanding and accepting things that are hard to accept or understand). Most of these aspects can be straightforwardly subsumed under the motive to strive for mastery. Explanation, however, goes somewhat beyond the mere instrumental domain by evoking the importance of meaning.

**Table 1:** Functional aspects of the belief – exemplary statements

Getting along with the world	
Orientation	“… the cards always told me … what my next path could be.” (B1, lines 10–14)
Coping with fear	“It started when I stopped being afraid. I used to be afraid of a lot of things […] the fear was gone.” (B11, lines 11–16)
Overcoming critical life events	“… my life can’t go on like this. And then I started looking for alternatives…” (B9, line 18).
Explanation	“Yes, because I noticed that […] there are things that cannot be explained. … like when people miraculously get better. Or when you suddenly know things that you shouldn’t know.” (B8, line 4).
Dealing with the self	
Caring for oneself	“And that […] was a moment of total clarity in my life. And just absolute mindfulness.” (B3, line 14).
Developing the self	“And that’s why […] for me it’s […] the measure of quality: can something be used as a tool to grow as a person, yes or no? Is it helpful for me and can it help me improve myself?” (B5, line 40).
Self-awareness	“I could […] write who I am and how I am. With all the facets of myself, with all the critique, the positive things, just everything. … I feel like I know myself better.” (B2, line 20).

The table contains subcategories of the third category (utility of belief) with quotations of representative examples

“Dealing with the self” was the second subcategory. Many utterances correspond to the motive of valuing me and mine. Participants report that their beliefs help them to care for themselves and develop themselves further with regard to perspective taking, personality, and spiritual competences (see repository for these additional subcategories). Again, we found evidence for the importance of meaning and understanding the self.

Notably, however, functional aspects in terms of the second motive, connectedness, were not expressed by the participants.

## Discussion

The results of our qualitative study indicate that paranormal beliefs are instrumental for achieving several goals. They may provide a plethora of trajectories to develop the self-concept and build a valuable perception of oneself. Altogether, we found preliminary evidence that paranormal beliefs may be functional with regard to two fundamental motives – striving for mastery and valuing me and mine.

Paranormal beliefs are, by definition, in opposition to empirical facts and scientific explanations of the world. Similar to religious beliefs, they tend be immunized against critical examination (Albert 1985). In accordance with the evolutionary approach to religion, paranormal beliefs could be explained in terms of biases resulting from suboptimal thinking (Atran 2002; Guthrie 1993; Haselton and Buss 2000). In line with this interpretation, there is evidence showing that believers in the paranormal tend to avoid analytic styles of thinking (e.g., Aarnio and Lindeman 2005; Gray and Gallo 2016), confuse ontological categories (e.g., animate and inanimate, Lindeman et al. 2015; see also Betsch et al. 2020), and have problems with formal reasoning (e.g., Betsch et al. 2020; Musch and Ehrenberg 2002; Rogers et al. 2009).

From such a point of view, however, one is inclined to neglect the other side of these beliefs. For the case of religious beliefs, Kanazawa (2015) developed a theory positing a higher order level of adaptivity. Although some beliefs might not survive critical testing, they may serve other functions. In his approach, he assumed that there is an adaptive link between (biased) beliefs and mood states. A positive mood state may have a halo effect on all aspects of adaptation including survival and re-production (Diener et al. 2015).

Our study supports the notion that paranormal beliefs can also be functional. The reports of our participants revealed an association between fundamental needs and paranormal beliefs. Yet, still there is a missing link. Believing, for instance, in ghosts, angels, or a universal life energy does not directly make us happier, give us a sense of mastery, or help us to value me and mine. The interviews with our participants showed that paranormal beliefs are embedded in rich and elaborated stories. *Homo sapiens* are the only animals who are capable of telling stories about things that cannot directly be observed or sensed (Harari 2015). What makes up a good story? Jerome Bruner (1993) identified at least two psychologically important features: creating meaning and providing pathways to the future. Meaning involves understanding. Pathways to the future refers the generic nature of good stories. Thus, stories can inform judgments, define criteria for decision making, and may provide strategies for problem solving. The stories told by our participants revealed elaborated networks connecting paranormal beliefs with the person’s biography. As such, insights and meaning about one own’s life can serve as a fundamental basis for making inferences, judgments, and decisions. Similar to Kanazawa’s theory on religious beliefs, paranormal beliefs may not have an adaptive value per se. Their *indirect* functional contribution may depend on the degree to which they help individuals form elaborated stories about their lives and their living. These stories may fulfill a plethora of functions. They appear to be instrumental for satisfying fundamental motivations, goal setting, and leading a meaningful life. With regard to their potentially indirect adaptive value, paranormal beliefs share characteristics of religious beliefs (Kanazawa 2015).

Although we found evidence for functional aspects of paranormal beliefs, they may at the same time have detrimental effects on adaptation. Gebauer et al. (2012) argued that the adaptive value of any belief cannot be universally defined in isolation to the social context. In Western societies, paranormal beliefs are prone to conflict with both dominant Christian religion and a scientific-rationalistic orientation. Idiosyncratic foundations of beliefs, e.g., encounters with ghosts, demons, or rebirthing experiences, may invite skeptical reactions in other people and may eventually result in social exclusion. Accordingly, our participants frequently reported that their resulting convictions were not regularly accepted by other people. Rather, their social environment often challenged their beliefs, provoking our participants to rely on strategies of immunization and demarcation to bolster their belief systems. Overall, paranormal beliefs appear to make individuals susceptible to challenges with regard to connectedness.

As already noted, a number of studies indicate that belief in the paranormal is negatively correlated with measures of systematic thinking. In a recent study with German participants, Hamdorf and Graf (2018) reported strong correlations between belief in the paranormal and science denial. On the other hand, paranormal beliefs were positively correlated with trust in dubious healing methods and negatively correlated with knowledge about them. This pattern of beliefs and attitudes might entail maladaptive health behavior, for example, opposing vaccinations or denying the effectiveness of anti-pandemic measures.

Our participants openly told us elaborate stories revealing diverse facets of their belief systems. A considerable number of those stories address functional aspects of how to get along with the world and deal with the self. Their beliefs appear to be essential in terms of identifying what might be conceived as the true basis of their lives. As such, paranormal beliefs may help the individual to make sense of their own biography. Thus, as a general theme that ties all the narratives together, one can describe the paranormal beliefs in terms as a generator of meaning and understanding. These beliefs yield subjective, generic theories of the world. Not only do they account for the past, they also provide orientation for goal setting and future behavior. Without a doubt, our qualitative study shares the obvious limitations of any small-sample research with minimal control over potentially confounding variables. It would not be justified to generalize our findings to other samples and contexts. The study’s value lies in making us aware of factors that, thus far, have been widely neglected in research on paranormality. Our participants spontaneously and eagerly addressed functional issues without being invited to do so. These findings should encourage researchers to acknowledge *adaptive mechanisms* when investigating the determinants of paranormal beliefs that are still widespread in society.

## Data Availability

https://osf.io/ck5xy/.

## References

[CR1] Aarnio K, Lindeman M (2005). Paranormal beliefs, education, and thinking styles. Personality and Individual Differences.

[CR2] Ajzen I, Fishbein M (1980). Understanding attitudes and predicting social behavior.

[CR3] Albert H (1985). Treatise on critical reason.

[CR4] American Psychiatric Association (1994). Diagnostic and statistical manual of mental disorders.

[CR5] Atran S (2002). In gods we trust: The evolutionary landscape of religion.

[CR6] Betsch T, Aßmann L, Glöckner A (2020). Paranormal beliefs and individual differences: Story seeking without reasoned review. Heliyon.

[CR7] Bruner J (1993). Acts of meaning – Four lectures of mind and culture.

[CR8] Dagnall N, Parker A, Munley G (2007). Paranormal belief and reasoning. Personality and Individual Differences.

[CR9] Diener E, Kanazawa S, Suh EM, Oishi S (2015). Why people are in a generally good mood. Personality and Social Psychology Review.

[CR10] Drinkwater K, Denovan A, Dagnall N, Parker A (2017). An assessment of the dimensionality and factorial structure of the revised paranormal belief scale. Frontiers in Psycholology.

[CR11] Festinger L (1954). A theory of social comparison processes. Human Relations..

[CR12] Gebauer JE, Sedikides C, Neberich W (2012). Religiosity, social self-esteem, and psychological adjustment: On the cross-cultural specificity of the psychological benefits of religiosity. Psychological Science.

[CR13] GESIS - Leibniz Institute for the Social Sciences. (2013). ALLBUS/GGSS 2012 (Allgemeine Bevölkerungsumfrage der Sozialwissenschaften/German General Social Survey 2012). GESIS Data Archive, Cologne. ZA4614 Data file Version 1.1.1. 10.4232/1.11753.

[CR14] Gray SJ, Gallo DA (2016). Paranormal psychic believers and skeptics: A large-scale test of the cognitive differences hypothesis. Memory & Cognition.

[CR15] Guthrie SE (1993). Faces in the clouds: A new theory of religion.

[CR16] Hamdorf, E., & Graf, D. (2018). Wissen und Einstellung zu Alternativmedizin [knowledge and attitudes towards alternative medicine]. *Skeptiker, 4*, 160–168.

[CR17] Harari YN (2015). Sapiens - a brief history of humankind.

[CR18] Haselton MG, Buss DM (2000). Error management theory: A new perspective on biases in cross-sex mind reading. Journal of Personality and Social Psychology.

[CR19] Henningsgaard J, Arnau R (2008). Relationships between religiosity, spirituality, and personality: A multivariate analysis. Personality and Individual Differences.

[CR20] Henrie J, Patrick JH (2014). Religiousness, religious doubt, and death anxiety. International Journal of Aging & Human Development.

[CR21] Hergovich A, Arendasy M (2005). Critical thinking ability and belief in the paranormal. Personality and Individual Differences.

[CR22] Houran J, Lange R (2004). Redefining delusion based on studies of subjective paranormal ideation. Psychological Reports.

[CR23] Irwin HJ (1992). Origins and functions of paranormal beliefs: The role of childhood trauma and interpersonal control. Journal of the American Society for Psychical Research.

[CR24] Irwin HJ (1994). Belief in the paranormal: A review of the empirical literature. Psychological Reports.

[CR25] James W (1902). The varieties of religious experience - a study in human nature.

[CR26] Kanazawa S (2015). Where do gods come from?. Psychology of Religion and Spirituality.

[CR27] Klaus, J. (2017). Wer am Geschäft mit dem Seelenheil verdient. *Süddeutsche Zeitung*. http://www.sueddeutsche.de/wirtschaft/esoterik-wer-am-geschaeft-mit-dem-seelenheil-verdient-1.3596195, retrieved Jan. 2019.

[CR28] Kruse J (2014). Qualitative Interviewforschung. Ein integrativer Ansatz [Qualitative interview research: An integrative approach].

[CR29] Kuckartz U, Dresing T, Rädiker S, Stefer C (2008). Qualitative evaluation. Der Einstieg in die praxis [Qualitative evaluation - introduction for practitioner].

[CR30] Kunda Z (1990). The case for motivated reasoning. Psychological Bulletin.

[CR31] Lange R, Houran J (1999). The role of fear in delusions of the paranormal. Journal of Nervous and Mental Disease.

[CR32] Lange R, Irwin HJ, Houran J (2000). Top-down purification of Tobacyk's revised paranormal belief scale. Personality and Individual Differences.

[CR33] Lindeman M, Svedholm AM (2012). What’s in a term? Paranormal, superstitious, magical and supernatural beliefs by any other name would mean the same. Review of General Psychology.

[CR34] Lindeman M, Svedholm-Häkkinen AM, Lipsanen J (2015). Ontological confusions but not mentalizing abilities predict religious belief, paranormal belief, and belief in supernatural purpose. Cognition.

[CR35] Loosen, W. (2014) Das Leitfadeninterview – eine unterschätzte Methode. In: Averbeck-Lietz S., Meyen M. (eds), Handbuch nicht standardisierte Methoden in der Kommunikationswissenschaft. Springer NachschlageWissen. Springer VS, Wiesbaden. 10.1007/978-3-658-05723-7_9-1

[CR36] Mayring P (2002). Einführung in die qualitative Sozialforschung [Introduction to social research].

[CR37] Mayring P, Fenzl T, Baur N, Blasius J (2014). Qualitative Inhaltsanalyse [Qualitative content analysis]. Handbuch Methoden der empirischen Sozialforschung [Manual of methods of empirical social research].

[CR38] McClelland DC (1987). Human motivation.

[CR39] McKay R, Dennett D (2009). The evolution of misbelief. Behavioral and Brain Sciences.

[CR40] Musch J, Ehrenberg K (2002). Probability misjudgment, cognitive ability, and belief in the paranormal. British Journal of Psychology.

[CR41] Potten, M., & Memminger, C. (2017). Esoterik für alle: Wie Ersatzreligionen an unser Geld kommen. http://www.daserste.de/information/wirtschaft-boerse/plusminus/esoterik-glaube-seele-sinnsuche-100.html, retrieved Jan. 2019.

[CR42] Rasmussen CH, Johnson ME (1994). Spirituality and religiosity: Relative relationships to death anxiety. OMEGA - Journal of Death and Dying.

[CR43] Risen JL (2016). Believing what we do not believe: Acquiescence to superstitious beliefs and other powerful intuitions. Psychological Review.

[CR44] Rogers P, Davis T, Fisk J (2009). Paranormal belief and susceptibility to the conjunction fallacy. Applied Cognitive Psychology.

[CR45] Schnell T (2012). Spirituality with and without religion—Differential relationships with personality. Archive for the Psychology of Religion.

[CR46] Schreier, M. (2014). Varianten qualitativer Inhaltsanalyse: Ein Wegweiser im Dickicht der Begrifflichkeiten. *FQS, 1.* http://www.qualitative-research.net/index.php/fqs/article/view/2043.

[CR47] Smith ER, Mackie DM (2000). Social psychology.

[CR48] Stamann, C., Janssen, M., & Schreier, M. (2016). Qualitative Inhaltsanalyse – Versuch einer Begriffsbestimmung und Systematisierung, *FQS, 3.* http://www.qualitative-research.net/index.php/fqs/issue/view/56.

[CR49] van Prooijen J-W, Douglas KM, De Inocencio C (2017). Connecting the dots: Illusory pattern perception predicts belief in conspiracies and the supernatural. European Journal of Social Psychology.

[CR50] Von Neumann J, Morgenstern O (1947). Theory of games and economic behavior.

[CR51] Williams E, Roberts BLH (2016). The relationship between paranormal belief and the Hexaco domains of personality. Journal of Empirical Theology.

[CR52] Wolfradt U (1997). Dissociative experiences, trait anxiety, and paranormal beliefs. Personality and Individual Differences.

